# Supplementary material for: Photoluminescence imaging of single photon emitters within nanoscale strain profiles in monolayer WSe2
Source: Nat Commun. 2023 Sep 15;14:5737. doi: 10.1038/s41467-023-41292-9 (PMC10504242; doi:10.1038/s41467-023-41292-9)
Supplement: Supplementary file 1 — Supplementary Information [file 41467_2023_41292_MOESM1_ESM.pdf]

Supplementary Information for: Photoluminescence imaging of single photon emitters within nanoscale strain profiles in monolayer WSe_2

Artem N. Abramov,[1] Igor Y. Chestnov,[1] Ekaterina S. Alimova,[2]
Tatiana Ivanova,[1] Ivan S. Mukhin,[1,3] Dmitry N. Krizhanovskii,[4]
Ivan A. Shelykh,[1,5,6,7] Ivan V. Iorsh,[1,6,7] and Vasily Kravtsov[1,*]

[1]*School of Physics and Engineering, ITMO University, Saint Petersburg 197101, Russia*

[2]*Peter The Great St. Petersburg Polytechnic University, Saint Petersburg 195251, Russia*

[3]*St. Petersburg Academic University, Saint Petersburg 194021, Russia*

[4]*Department of Physics and Astronomy, University of Sheffield, Sheffield S3 7RH, UK*

[5]*Science Institute, University of Iceland, Dunhagi-3, IS-107 Reykjavik, Iceland*

[6]*Abrikosov Center for Theoretical Physics, MIPT, Dolgoprudnyi, Moscow Region 141701, Russia*

[7]*Russian Quantum Center, Skolkovo, Moscow 143025, Russia*

Supplementary Note 1: Probes for nanoindentation

In order to form nanoindents in WSe_2 monolayers, we used modified commercial silicon atomic force microscopy (AFM) probes (model VIT_P from NT-MDT Spectrum Instruments). We modified each probe by blunting its apex with Ga focused ion beam (FIB) using Zeiss Auriga electron–ion microscope with accelerating voltage of 30 kV and current of 1 pA. We found that the most reproducible SPEs are formed when using a probe with the lateral size of the blunted apex of 500 nm. This is likely due to the fact that stresses in WSe_2 monolayer appear in a sufficiently large area where defects are located. On the contrary, when using an unmodified probe, we were unable to form emitters, most likely due to premature rupturing of the monolayer. Supplementary Figure 1 shows scanning electron microscope (SEM) images of a selected probe before (a) and after (b) modification.

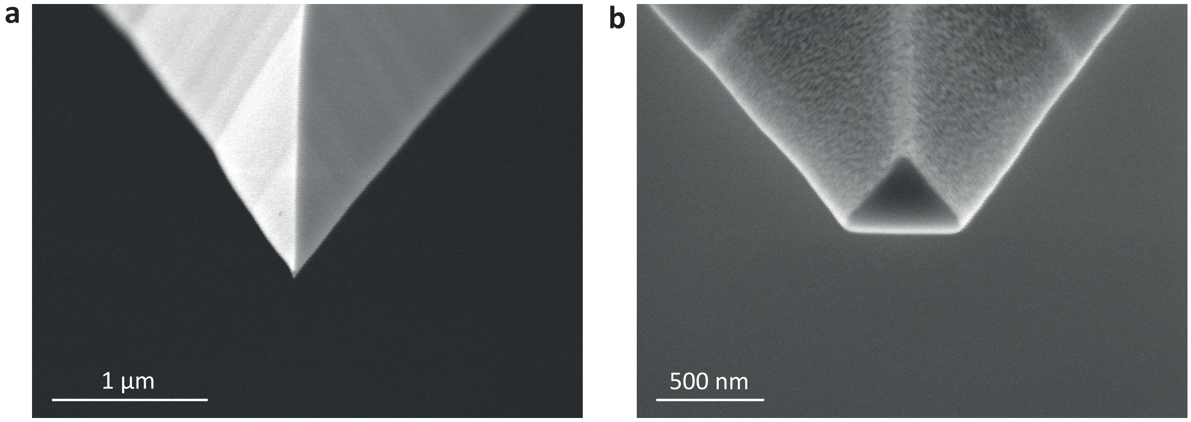

Supplementary Figure 1. SEM image of a selected AFM probe used for nanoindentation in our experiment before modification with Ga FIB (a) and after modification (b).

Our nanoindentation method allows precise positioning of the tip with respect to a single defect with known coordinates, which could in principle enable investigating the role of the relative position between the defect and the induced strain profile. However, we know the coordinates of the defect only after we locally strain the monolayer via deforming the polymer substrate in an irreversible way. In experiments, we observe that further attempts to create locally strained regions within 1 μm distance of the original indent result in the disappearance of single-photon emission from existing SPEs. This agrees with the expectations from our theoretical model, since any further deformations in the vicinity of the original strained region will likely change the local strain tensor components at the defect location

and bring the dark exciton out of resonance with the defect energy level.

Supplementary Note 2: Optical setup

Supplementary Figure 2 shows a schematic of the optical setup used in the experiment. Setup allows obtaining an image of the sample on a CMOS camera while illuminated with two light sources simultaneously. The sample is placed in a closed-loop helium cryostat with base temperature of 6 K. Photoluminescence (PL) was excited by a HeNe laser with 633 nm wavelength. A halogen lamp was used to illuminate the alignment marks. To do this, a 10 nm wide line in the long-wavelength range with an energy less than the energy of excitons and trions in WSe_2 monolayer was cut out of the lamp spectrum using a bandpass filter in order to avoid excitation of PL. Excitation and detection was performed with a 50× microscope objective (NA = 0.65) Mitutoyo M Plan Apo NIR HR 50X. Laser light was focused on the surface of the sample to a point with a diameter of ~ 1.5 µm. Light of the lamp was focused in the back focal plane of the objective to achieve uniform illumination of the entire surface of the sample. The objective was mounted on a piezoelectric translator to enable positioning the focused laser spot on a specific indent.

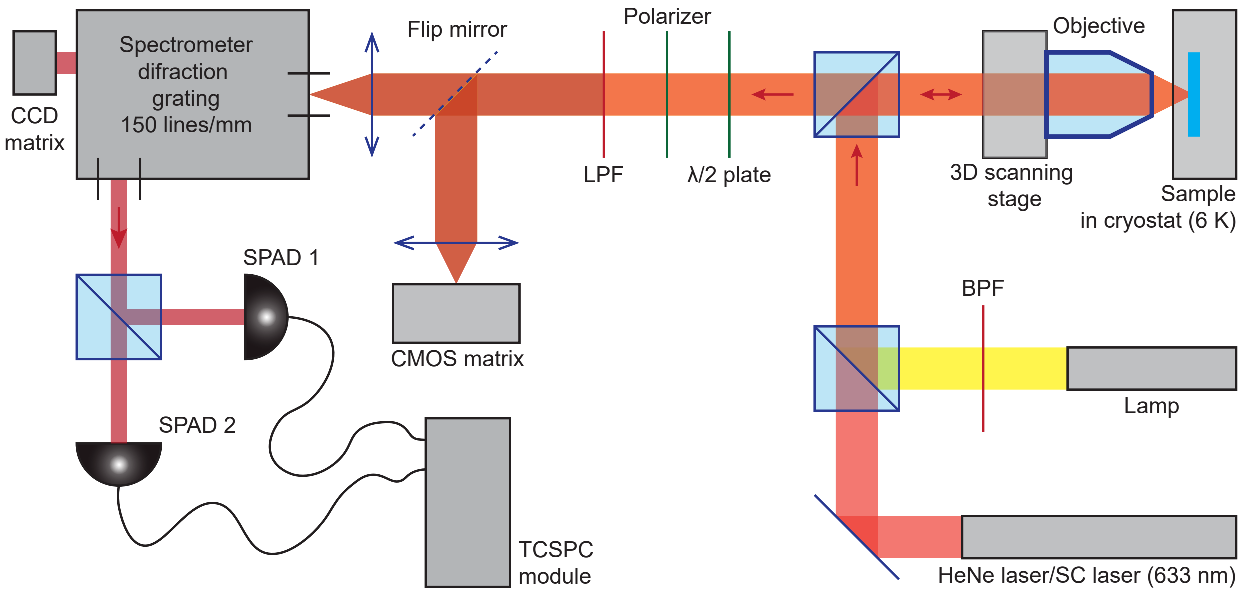

Supplementary Figure 2. Schematic of the optical setup used in the experiment. LPF: long pass filter, BPF: band pass filter, SC laser: supercontinuum laser, TCSPC: time correlated single photon counting.

PL of the monolayer was selected with a 750 nm long pass filter. To investigate the polarization properties of the SPEs, we used a polarizer and a half-wave plate installed on a motorized rotator. The PL spectra were measured with a Princeton Instruments Acton SP-2500 spectrometer with 150 lines/mm grating and liquid nitrogen cooled CCD camera. Individual emission lines from SPEs filtered by the spectrometer were studied with a Hanbury Brown and Twiss setup consisting of a 50/50 beamsplitter, two single-photon detectors SPCM-AQRH-W4, and time-correlated single photon counting (TCSPC) module PicoHarp300. For measurements of PL lifetime we used excitation with a supercontinuum laser (WhiteLase SC400, Fianium) with repetition rate of 60 MHz and SuperChrome filter at 633 nm wavelength.

Supplementary Note 3: Polarization filtering

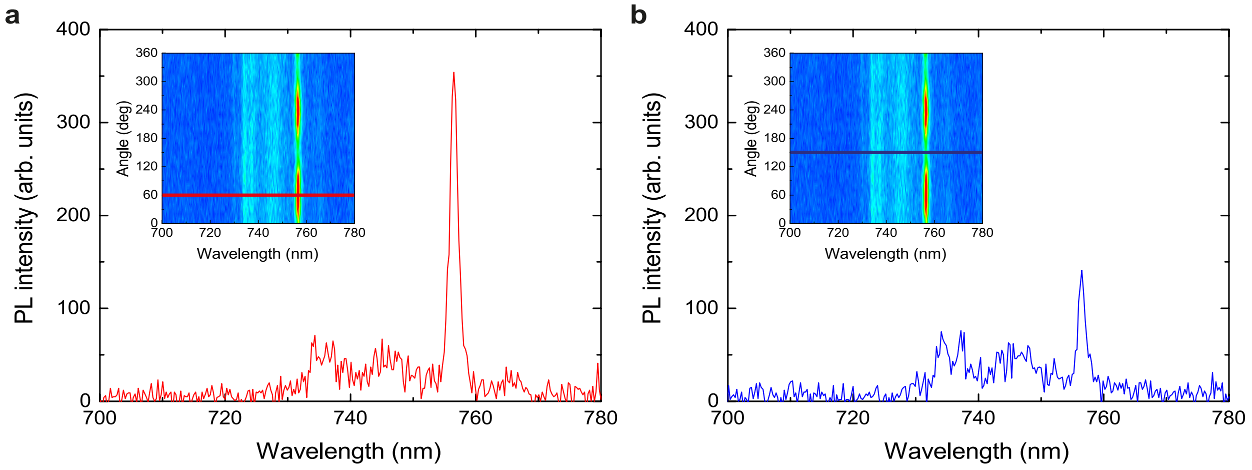

Supplementary Figure 3. (a) PL spectrum at the polarization direction corresponding to the maximum intensity of the SPE peak; inset shows the dependence of PL spectrum on the polarization direction. (b) PL spectrum at the polarization direction corresponding to the minimum intensity of the SPE peak; inset shows the full dependence on the polarization direction with the black line indicating the selected angle. At the same time, the signal of the background is mostly unpolarized.

The SPEs created in our experiment demonstrate predominantly linear polarization of radiation. Supplementary Figure 3 shows PL spectra obtained from a single nanoindent in co and cross polarization relative to the polarization angle of the SPE peak at 756 nm without normalization. The insert shows the dependence of PL spectrum on polarization

direction. The unpolarized emission of the background and the linearly polarized peak of the quantum emitter are observed in the maps. The high degree of polarization of single photon sources allowed us to increase the ratio of the useful signal to noise ratio.

Supplementary Note 4: Brightness characteristics of single photon sources

To estimate the emission rate achievable with our SPEs, we measured dependencies of the SPE PL intensity on the excitation laser power. We fitted the experimental dependencies of the integral PL intensity on the pump power ($I(P)$) using the equation for a two-level system $I(P) = I_0/(1+P_{sat}/P)$, where P is pump power, I_0 is the maximum SPE PL emission rate, and P_{sat} is saturation power (I_0 and P_{sat} are fit parameters). From the fitting equation, we extracted the saturation power, which lies in a $0.7-1.0$ μW range for our emitters. A typical curve of SPE PL emission rate for one of the emitters is shown in Supplementary Figure 4a (red squares) together with corresponding data for the neutral exciton PL (blue triangles). The observed saturation behavior (red curve) additionally confirms the single-emitter origin of the PL signal. In contrast, the neutral exciton PL demonstrates a linear dependence on the excitation power (blue line).

To estimate emission rates, we converted experimental values of PL intensity of our SPEs from counts on the detector to the number of emitted photons per second. The SPE PL emission rate was calculated from the photon count rate measured by a single-photon detector using the formula $N_{real} = (N*CF-DC)*k$, where N is photon count rate measured by the detector, CF is the correction factor for the detector, DC is dark count rate of the detector, and k is the coefficient accounting for the transmission in our optical setup and quantum efficiency of the detector. We present brightness statistics for our single photon sources in Supplementary Figure 4b. We also estimated the PL quantum yield for these single photon sources. Single photon source was pumped by a pulsed laser with 60 MHz frequency and wavelength of 633 nm with pump power close to the saturation power of the emitter. It was assumed that each laser pulse excites the emitter into a non-equilibrium state with subsequent relaxation. The internal PL quantum yield was estimated by the formula $q = N_{real}/f$, where q is PL quantum yield, N_{real} is brightness and f is laser pulse frequency. We demonstrate quantum yield statistics for our SPEs in Supplementary Figure 4c.

Additionally, we note that the brightness characteristics of the studied emitters can de-

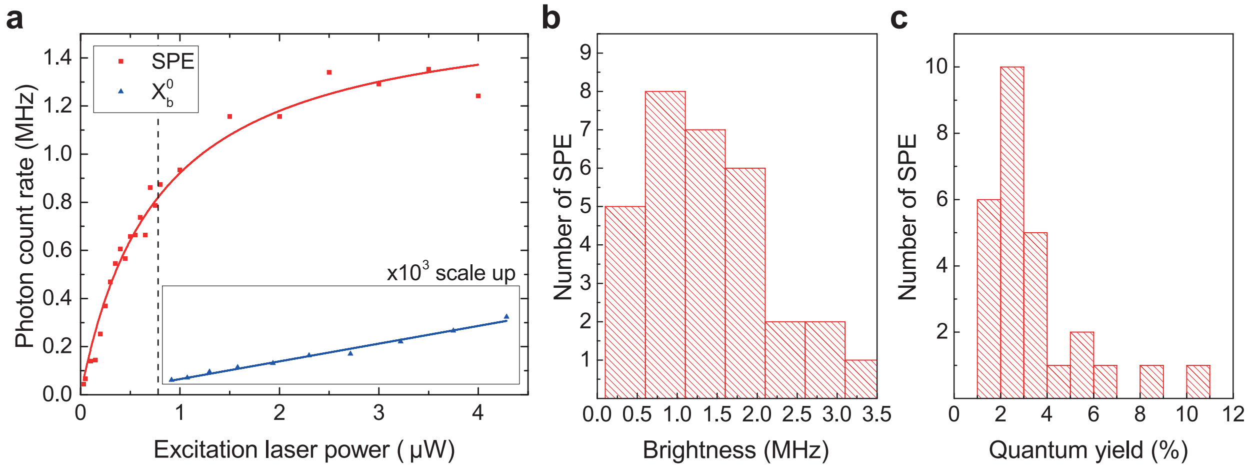

Supplementary Figure 4. (a) Dependence on excitation power for PL intensity of a selected SPE (red dots are experimental curve, red line is the fit function) and neutral exciton X_b^0 (blue triangles are experimental curve, blue line is linear fit function) in a WSe_2 monolayer. The vertical line indicates the saturation power extracted from the fit and equal to 0.78 μW. Pumping was carried out by 633 nm HeNe laser. (b) Brightness statistics for 31 SPEs. (c) PL quantum yield statistics for 31 SPEs.

grade with time. The PL spectra measured on our experimental samples are generally reproducible within $2-3$ thermal cycles. After that, we start to observe some degradation of the SPE brightness accompanied with nm-scale shifts of the emission wavelength. We believe that the slow changes of the emission characteristics are due to laser-induced heating of the locally strained regions, since the effect is usually more pronounced for measurements with higher excitation power.

Supplementary Note 5: Lifetime and $g^{(2)}$ measurements

We study the single-photon character of the emitted PL by measuring the second-order autocorrelation $g^{(2)}(\tau)$ as a function of time delay τ. We process the data with $g^{(2)}(\tau) = 1-(1-A)\,e^{-|\tau/\tau_0|}$ for a two-level system or $g^{(2)}(\tau) = 1-(1-A+C)\,e^{-|\tau/\tau_0|} + C\,e^{-|\tau/\tau_0|}$ for a three-level system when measured in saturation mode (A, C and τ_0 are fit parameters). All data has been processed without correction for the background and dark counts of the detectors. While in the main text we show the best achieved value of $g^{(2)}(0) = 0.02$, typical $g^{(2)}(0)$ vary from SPE to SPE and are generally below 0.2. Supplementary Figure 5a

shows a second-order correlation function for one of the SPE and demonstrates the value of $g^{(2)}(0) \simeq 0.07 \pm 0.04$ and value of $\tau_0 = 4.8$ ns. We present our $g^{(2)}(0)$ statistics data in Supplementary Figure 5b with the average value of $g^{(2)}(0)$ of 0.15. Supplementary Figure 5c shows all values of $g^{(2)}(0)$ with corresponding error bars obtained by fitting.

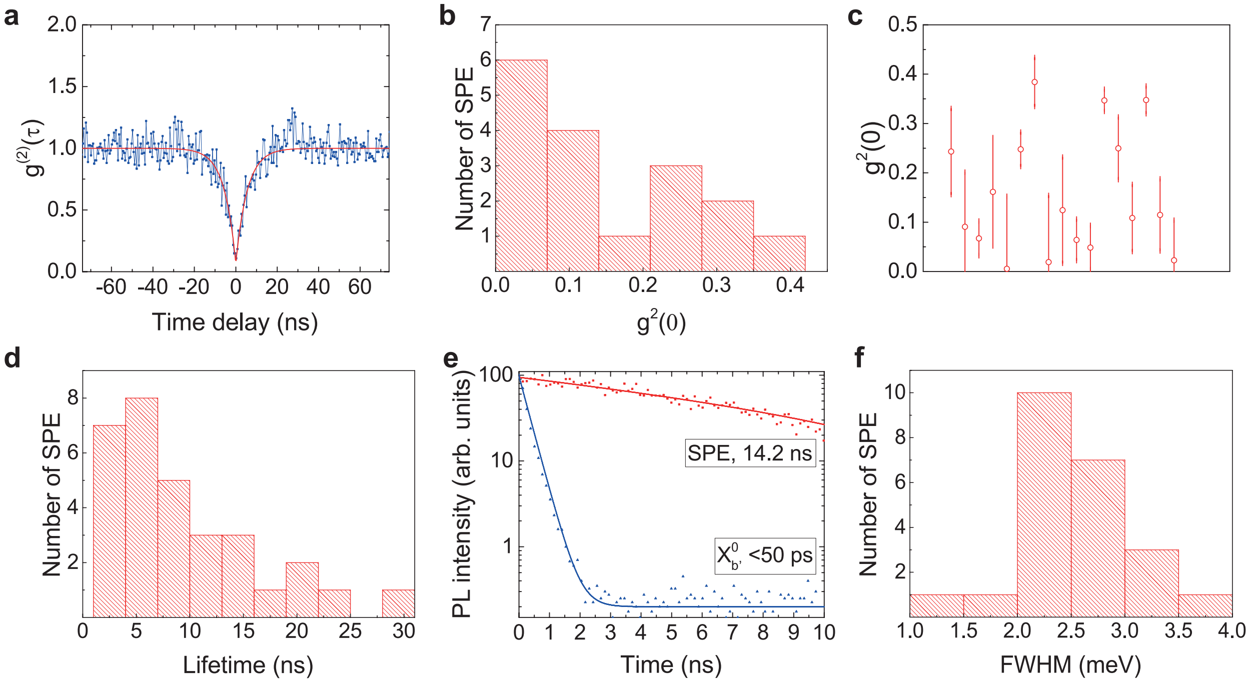

Supplementary Figure 5. (a) $g^{(2)}(\tau)$ function for a selected SPE, with blue dots corresponding to the experimental data points and red line corresponding to the fit. (b) $g^{(2)}(0)$ statistics for 17 SPEs. (c) $g^{(2)}(0)$ values with error bars. (d) Lifetime statistics for 31 SPEs. (e) PL decay curve for the same SPE, with red dots corresponding to the experimental data points and red line corresponding to the fit, and PL decay curve for the neutral exciton X_b^0 in the WSe_2 monolayer (blue triangles are experimental data, and blue curve is a fit). (f) FWHM statistics for 23 SPEs.

In addition, we investigated the PL decay times for the studied SPEs, with a significant spread of lifetime values in the range from 1.5 to 30.8 ns and the average value is about 10 ns (Supplementary Figure 5d). Supplementary Figure 5e shows the PL decay time for selected SPE with a characteristic value 14.2 ns and for neutral exciton X_b^0 in the WSe_2 monolayer with the value of less than 50 ps (limited by the resolution of our experimental setup). We note that the corresponding radiative linewidth values are < 1 μeV, while the FWHM of the measured SPE peaks is on the order of 2 meV (statistics data in Supplementary Figure 5f),

which likely includes contributions due to spectral wandering and pure dephasing related to interaction with surroundings. In order to achieve generation of indistinguishable photons in the future, the linewidth should be reduced significantly, and Purcell enhancement with factors on the order of 100-1000 are desirable.

Supplementary Note 6: Additional measurements of SPE site locations

The results of PL imaging for different SPEs show that the SPE sites are located near the rim formed around the nanoindent and generally on its outer side. In Supplementary Figure 6 we show data for three additional studied emitters formed at deformed regions made with slightly different indentation depth values. Similar to the data presented in the main text, here we show AFM topography maps for each nanoindented region overlaid with the extracted SPE positions indicated with white crosses. The X and Y arms of the crosses correspond to the experimental error bars obtained for the X and Y coordinates. As observed in Supplementary Figure 6, emitters can be formed at different sides of the indented region. On the other hand, we did not observe correlation between the SPE properties and indentation depth for depth values in the range from 100 nm to 200 nm.

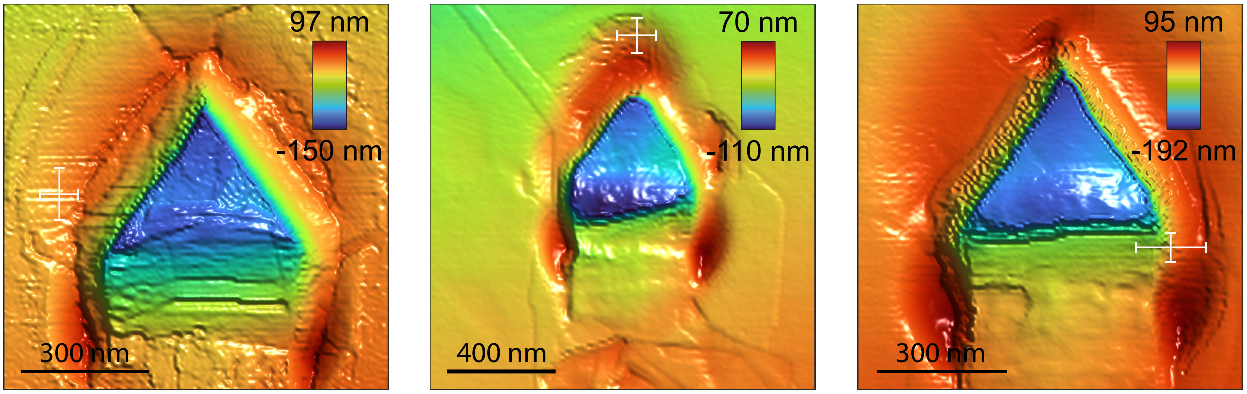

Supplementary Figure 6. Extracted positions for additional SPE sites: AFM topography of the strained regions (false color maps) overlaid with experimentally extracted SPE positions indicated with crosses. Experimental uncertainties for extracted SPE coordinates are indicated with X and Y error bars.

Supplementary Note 7: Calculation of the strain distribution

In this supplementary section we describe the procedure of extracting the in-plane strain distribution from the height profile obtained from AFM measurements.

Let us consider a 2D crystal subject to a permanent deformation. An external load applied from the top crumples the substrate layer of PMMA. The monolayer loosely covers the deformed surface. The resulting out-of-plane deformation profile $h(x, y)$ is sufficiently smooth on the inter-atomic distance scale. That is why, we take advantageous of the continuous elasticity theory and consider the monolayer as a plate with infinitesimally small thickness. The strain appeared in response to the deformation is thus characterised by the position-dependent second-rank tensor $\hat{\epsilon}(\boldsymbol{r})$, where $\boldsymbol{r}$ defines the in-plane position vector. In the linear limit, Cartesian components of the strain tensor can be connected with the local displacements as [1]

$$\epsilon_{ij} = \frac{1}{2}\left(\partial_i u_j + \partial_j u_i + \partial_i h \partial_j h\right), \tag{1}$$

where $u_x(\boldsymbol{r})$ and $u_y(\boldsymbol{r})$ are the in-plane displacements along x and y-directions, respectively. Although the $\boldsymbol{u}(\boldsymbol{r})$-distribution is unknown, it can be directly connected with the height profile $h(\boldsymbol{r})$. Indeed, due to the finite resistance to strain, shifting a monolayer in the out-of-plane direction entails in-plane displacements as it is illustrated in Supplementary Figure 7a. The equilibrium configuration of the displacement field $\boldsymbol{u}(\boldsymbol{r})$ must minimize the classical elastic energy. Alternatively, one can resort to the in-plane force equilibrium condition which is formulated in terms of the stress (force per area) tensor $\hat{\sigma}$:

$$\partial_x \sigma_{xx} + \partial_y \sigma_{xy} = 0, \tag{2a}$$

$$\partial_x \sigma_{xy} + \partial_y \sigma_{yy} = 0, \tag{2b}$$

or in short notations $\hat{\sigma} \cdot \boldsymbol{\nabla} = 0$ with $\boldsymbol{\nabla} = (\partial_x, \partial_y)^\intercal$. Eqs. (2) ensure that equilibrium of the material is maintained at any position $\boldsymbol{r}$. The local stress $\hat{\sigma}$ can be connected with strain tensor components using the Hook's law (Eq. (2) in the manuscript),

$$\begin{pmatrix} \sigma_{xx} \\ \sigma_{yy} \end{pmatrix} = \frac{E}{1-\nu^2} \begin{pmatrix} 1 & \nu \\ \nu & 1 \end{pmatrix} \begin{pmatrix} \epsilon_{xx} \\ \epsilon_{yy} \end{pmatrix}, \qquad \sigma_{xy} = \frac{E}{1+\nu}\epsilon_{xy}. \tag{3}$$

which defines the isotropic elastic parameters of the monolayer which are the Young's modulus E and the Poisson's ratio ν.

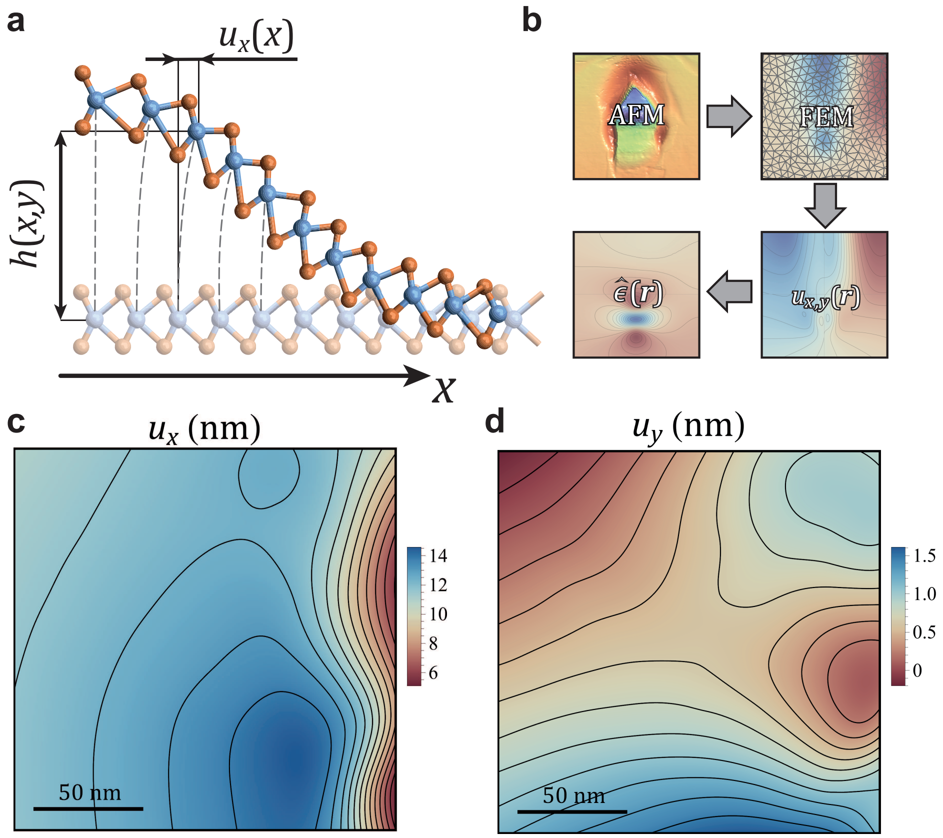

Supplementary Figure 7. The strain calculation procedure. (a) The sketch of the deformed monolayer. The out-of-plane shift $h(\boldsymbol{r})$ leads to the in-plane displacement of the monolayer elements. (b) The block-diagram illustrating the operating sequence for the strain tensor calculation. (c) and (d) the displacement field components $u_x(\boldsymbol{r})$ and $u_y(\boldsymbol{r})$ which correspond to the strain profile shown in Fig. 3b and c in the manuscript.

Substituting Eqs. (1) and (3) into (2) yields two coupled equations for the in-plane displacement components:

$$\partial^2_{xx}u_x + \frac{1-\nu}{2}\partial^2_{yy}u_x + \frac{1+\nu}{2}\partial^2_{xy}u_y + \partial_x h\left(\partial^2_{xx}h + \frac{1-\nu}{2}\partial^2_{yy}h\right) + \frac{1+\nu}{2}\partial_y h\partial^2_{xy}h = 0, \quad (4a)$$

$$\partial^2_{yy}u_y + \frac{1-\nu}{2}\partial^2_{xx}u_y + \frac{1+\nu}{2}\partial^2_{xy}u_x + \partial_y h\left(\partial^2_{yy}h + \frac{1-\nu}{2}\partial^2_{xx}h\right) + \frac{1+\nu}{2}\partial_x h\partial^2_{xy}h = 0. \quad (4b)$$

With the given height profile $h(\boldsymbol{r})$ extracted from the AFM data, these equations can be solved with the finite-element method (FEM). Then, using the in-plane displacement field $\boldsymbol{u}(\boldsymbol{r})$ the full strain tensor $\hat{\epsilon}(\boldsymbol{r})$ can be reconstructed from the definition (1). The examples of the extracted displacement fields $u_x(\boldsymbol{r})$ and $u_y(\boldsymbol{r})$ are shown in Supplementary Figure 7c

and d. These data correspond to the local strain distribution and the strain-asymmetry map presented in Fig. 3b and c in the main manuscript.

The calculation procedure is schematically illustrated in Supplementary Figure 7b. Note that Eqs. (4) do not contain the Young modulus E. Therefore, the Poisson's ratio ν is the only material parameter used in calculations. We take $\nu = 0.196$ which is consistent with calculations and experimental data [2, 3].

Supplementary Note 8: Hybridization analysis of multiple SPEs

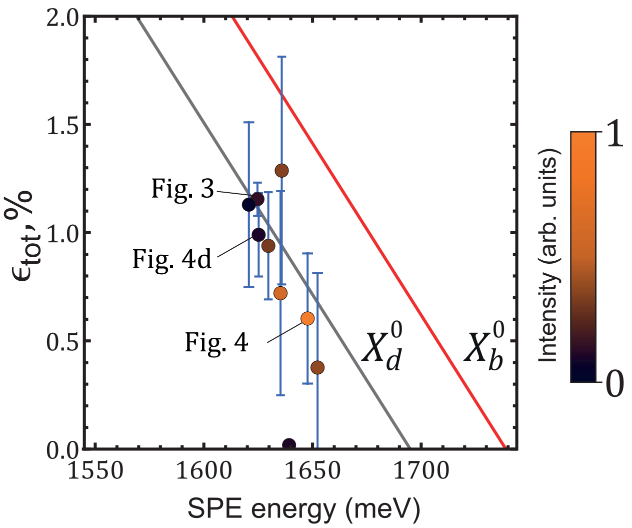

Supplementary Figure 8. The scatter plot of the calculated strain at the SPE position vs SPE emission energy. The vertical bars correspond to the mean-square deviation of the extracted strain distribution within the SPE position uncertainty domain. The color of the dot corresponds to the SPE emission intensity normalized to the maximal intensity value within the given set of SPEs. The inclined grey and red lines correspond to the strain-dependent spectral position of the dark X_d^0 and bright X_b^0 excitons, respectively.

The results of the strain analysis for several SPEs are summarised in Supplementary Figure 8. The calculated exciton energy shift at the SPE position plotted in units of the total strain $\epsilon_{\rm tot}$ on the vertical axis while the expected spectral positions of the dark and bright excitons as a function of strain are shown in grey and red, respectively. The detected emission energy of the most of the investigated SPEs lies close to the dark exciton resonance shifted in the presence of the local strain experienced by the given SPE. These results

further confirm the strain-induced hybridization hypothesis of the SPE origin. Note that the emitters with the highest brightness was observed near 1.64 meV which corresponds to the energy of the single Se-vacancy of WSe_2 monolayer estimated in [4]. The resonance between the dark exciton and the point-defect provides the best conditions for hybridization which maximizes SPE emission rate.

---

* Corresponding author: vasily.kravtsov@metalab.ifmo.ru

Supplementary References

[1] Landau, L. D., , Lifshitz, E. M., Kosevich, A. M. & Pitaevskii, L. P. Theory of elasticity (Elsevier, 1986).

[2] Zeng, F., Zhang, W.-B. & Tang, B.-Y. Electronic structures and elastic properties of monolayer and bilayer transition metal dichalcogenides MX_2 (M=Mo, W; X=O, S, Se, Te): a comparative first-principles study. Chin. Phys. B **24**, 097103 (2015).

[3] Blundo, E., Cappelluti, E., Felici, M., Pettinari, G. & Polimeni, A. Strain-tuning of the electronic, optical, and vibrational properties of two-dimensional crystals. Appl. Phys. Rev. **8**, 021318 (2021).

[4] Hernández López, P. et al. Strain control of hybridization between dark and localized excitons in a 2D semiconductor. Nat. Commun. **13**, 7691 (2022).
